# Combined Heart-Liver Transplantation: A First Successful Case in Chile

**DOI:** 10.7759/cureus.100116

**Published:** 2025-12-26

**Authors:** Cristobal Vildosola, Jhon W Arenas, Agustín Laclote, Pedro Becker, Eduardo Briceño, Rodrigo González, Loreley P Bermúdez, José T Peña, Douglas Greig, Carlos E Benitez, Ricardo Rojas, Jorge A Martínez

**Affiliations:** 1 School of Medicine, Pontificia Universidad Católica de Chile, Santiago, CHL; 2 Department of Cardiology, School of Medicine, Pontificia Universidad Católica de Chile, Santiago, CHL; 3 Section of Cardiovascular Surgery, Division of Surgery, School of Medicine, Pontificia Universidad Católica de Chile, Santiago, CHL; 4 Department of Digestive Surgery, School of Medicine, Pontificia Universidad Católica de Chile, Santiago, CHL; 5 Transplant Institute, School of Medicine, Pontificia Universidad Católica de Chile, Santiago, CHL; 6 Department of Pathology, School of Medicine, Pontificia Universidad Católica de Chile, Santiago, CHL; 7 Department of Gastroenterology, School of Medicine, Pontificia Universidad Católica de Chile, Santiago, CHL; 8 Department of Neurosurgery, School of Medicine, Pontificia Universidad Católica de Chile, Santiago, CHL

**Keywords:** combined heart-liver transplantation, heart transplantation, immunosuppression, liver transplantation, multi-organ failure

## Abstract

Combined heart-liver transplantation (CHLT) is a rare and highly complex procedure reserved for patients with end-stage failure of both organs. We report the first successful CHLT performed in Chile, involving a 55-year-old man with severe dilated cardiomyopathy and chronic liver disease who underwent sequential bicaval heart transplantation followed by piggyback liver transplantation. The postoperative course was complicated by acute neurological deterioration due to an acute subdural hematoma with tentorial extension requiring decompressive craniectomy, after which the patient recovered fully. More than one year after transplantation, he remains in excellent health, physically active, and fully reintegrated into work, with excellent function of both grafts. This case highlights the feasibility of CHLT in Chile and underscores the importance of multidisciplinary evaluation and coordinated perioperative management in optimizing outcomes for patients with advanced multi-organ failure.

## Introduction

Combined heart-liver transplantation (CHLT) has emerged as a definitive treatment for selected patients with concurrent end-stage cardiac and hepatic failure, particularly when isolated organ transplantation would not ensure adequate recovery [1-3]. Since the first successful CHLT reported in 1984 in a child with homozygous familial hypercholesterolemia, the procedure has steadily increased worldwide, with more than 400 cases documented in the United States by 2022 [1]. Over time, refinements in patient selection, perioperative management, and surgical technique have led to significantly improved outcomes, with current survival rates approximating those seen with isolated heart or liver transplantation [4,5].

The indications for CHLT have broadened substantially. The procedure is now considered in patients with a spectrum of cardiac and hepatic conditions, including familial hypercholesterolemia, severe cardiomyopathies with portal hypertension, congenital heart disease-related cirrhosis, restrictive and dilated cardiomyopathies, and metabolic diseases, including amyloidosis [6-9]. In such settings, cardiac dysfunction and hepatic dysfunction often progress in parallel, and single-organ transplantation may fail to provide meaningful recovery due to persistent hepatic dysfunction and portal hypertension, making CHLT the preferred strategy in carefully selected cases.

Despite increasing global experience, CHLT remains uncommon in Latin America. Only a handful of successful cases have been reported in Argentina and Brazil [6-8], highlighting the logistical, surgical, and organizational demands associated with establishing such programs. Chile has achieved significant advances in isolated heart and liver transplantation over the past decades [2,3], stabilizing the team's expertise to develop more complex multi-organ procedures. Experience with other combined procedures, such as liver-kidney transplantation, has been reported in Chile and across Latin America [10,11], demonstrating the capacity to perform complex multi-organ transplants in the region.

In this context, we report the first successful CHLT performed in Chile, carried out at the Hospital Clínico of the Pontificia Universidad Católica de Chile - Red Salud UC Christus. This case illustrates not only the feasibility of implementing CHLT in our national setting but also the multidisciplinary coordination required for optimal outcomes.

## Case presentation

A 55-year-old man with cryptogenic chronic liver disease (Child-Pugh B, 7 points, MELD-Na 24 points) and esophageal varices initially presented to his local hospital with progressive dyspnea, fatigue, and recurrent episodes of clinical decompensation. Initial evaluation revealed pulmonary edema and global systolic dysfunction, without evidence of pulmonary embolism.

**Figure 1:**
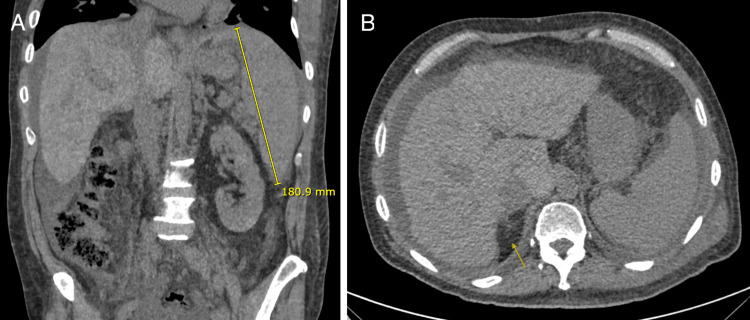
Abdominal CT scan (A) Coronal abdominal CT image demonstrating splenomegaly, with a maximal craniocaudal splenic length of 180.9 mm (normal adult splenic length ≤120–130 mm), consistent with clinically significant portal hypertension. (B) Axial abdominal CT image showing morphological features of chronic liver disease, including right hepatic lobe atrophy with a characteristic notch sign (arrow), defined as an indentation along the right hepatic contour reflecting segmental volume loss. Relative hypertrophy of the left lateral segment and caudate lobe, as well as a nodular hepatic contour, is also evident. Associated perihepatic and perisplenic ascites further support the diagnosis of portal hypertension.

Cardiac and hepatic evaluations were performed through multiple imaging studies at the main hospital of his region. Abdominal CT was consistent with chronic liver disease, including splenomegaly and ascites (Figure 1). These findings, together with recurrent hemodynamic decompensations, led to his referral to our transplant center in Santiago by plane to overcome 1,336 kilometers of distance for assessment to indicate a CHLT.

Upon admission to our institution, he presented with end-stage heart failure (LVEF 12% on SPECT-MIBI), secondary to idiopathic dilated cardiomyopathy, atrial flutter, and chronic liver disease with portal hypertension. He was evaluated by a multidisciplinary transplant committee and listed for CHLT.

During his prolonged hospitalization before transplant, he developed moderate pulmonary hypertension (pulmonary vascular resistance 4-5 Wood units) with a favorable vasodilator response. Progressive clinical deterioration led to initiation of percutaneous femoro-femoral venoarterial ECMO, mainly to assess organ recovery, particularly renal function, which had required ultrafiltration, and to determine candidacy for ongoing advanced support. A compatible donor was identified after 12 weeks of listing. Pre-transplant laboratory values are shown in Table 1.

**Table 1:** Pre-transplant laboratory tests PaO₂: arterial oxygen partial pressure; SaO₂: arterial oxygen saturation; PaCO₂: arterial carbon dioxide partial pressure; BUN: blood urea nitrogen; AST: aspartate aminotransferase; ALT: alanine aminotransferase; GGT: gamma-glutamyl transferase; INR: international normalized ratio; aPTT: activated partial thromboplastin time; eGFR: estimated glomerular filtration rate; MCV: mean corpuscular volume; MCH: mean corpuscular hemoglobin; RDW: red cell distribution width; CA 19-9: carbohydrate antigen 19-9; Pro-BNP: pro-B-type natriuretic peptide.

Parameter	Value	Reference Range
pH	7.45	7.35 – 7.45
BUN (mg/dL)	72.0	8 – 25
Urea (g/L)	1.54	0.17 – 0.54
Glucose (mg/dL)	134.0	70 – 99
Hemoglobin (g/dL)	7.7	12.0 – 16.0
Hematocrit (%)	21.4	36.0 – 46.0
AST (U/L)	48.0	≤ 35
ALT (U/L)	23.0	≤ 35
GGT (U/L)	137.0	≤ 40
Alkaline phosphatase (U/L)	159.0	30 – 100
Total bilirubin (mg/dL)	1.07	≤ 1.0
Direct bilirubin (mg/dL)	0.6	≤ 0.3
INR	1.3	0.8 – 1.2
Prothrombin time (%)	68.0	70 – 120
aPTT (seconds)	31.0	25 – 37
Albumin (g/dL)	4.8	3,4 a 5,4
Creatinine (mg/dL)	4.14	0.50 – 0.90
Estimated GFR (mL/min/1.73 m²)	15.0	≥ 90
CA 19-9 (U/mL)	5.9	≤ 34.0
Platelets (10³/µL)	102.0	140 – 400
Leukocytes (10³/µL)	4.7	4.5 – 11.0
Red blood cells (10⁶/µL)	2.46	4.00 – 5.20
MCV (fL)	86.9	80.0 – 100.0
MCH (pg)	31.3	26.0 – 34.0
RDW (%)	16.8	11.6 – 14.6

The combination of severe ventricular dysfunction, advanced portal hypertension, and progressive renal impairment, reflected by markedly reduced estimated glomerular filtration rate and the need for renal replacement therapy, was considered incompatible with isolated heart transplantation, supporting the initial indication for CHLT.

Surgical procedure

A standard brain death donor, a 31-year-old male with hypoxic-ischemic encephalopathy, was offered. Separate teams procured both organs. Under cardiopulmonary bypass, the recipient's heart was excised, and a bicaval heart transplant was performed. Total cardiopulmonary bypass time was 227 minutes; cold ischemia time for the heart was 2 hours and 29 minutes.

After confirming excellent cardiac function, the patient was decannulated, and the sternotomy was temporarily left open. The liver transplant team proceeded with a modified piggyback liver transplant technique and latero-lateral cavo-caval anastomosis [5]. The cold ischemia time of the liver graft was six hours and 26 minutes. Portal vein anastomosis was performed with subsequent portal reperfusion, followed by both arterial and biliary anastomoses. Thoracic and abdominal hemostasis was achieved, and the sternotomy and laparotomy were closed. The patient was transferred to the ICU in stable condition.

Postoperative course

Induction immunosuppression included methylprednisolone and basiliximab, followed by prednisone, mycophenolate, and tacrolimus. Initial support required multiple inotropes (milrinone, vasopressin, epinephrine, norepinephrine) and inhaled nitric oxide; all were discontinued by the 13th postoperative day.

**Figure 2:**
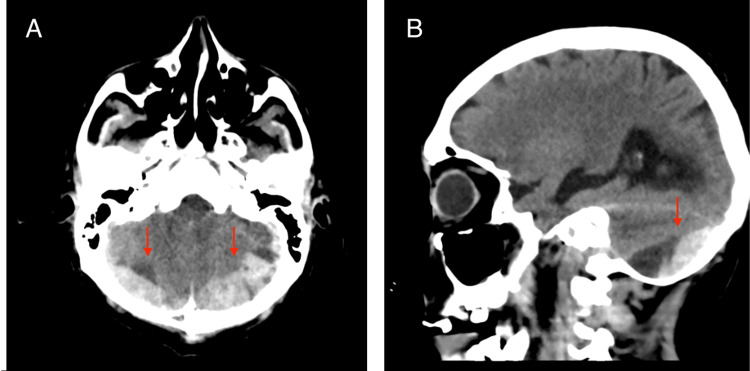
Brain CT scan (A) Axial brain CT image showing bilateral acute infratentorial subdural hematomas (arrows) with tentorial extension, resulting in significant posterior fossa mass effect. (B) Sagittal brain CT image demonstrating compression of the fourth ventricle and brainstem displacement, consistent with obstructive hydrocephalus. These findings correlated with acute neurological deterioration and prompted emergent neurosurgical decompression.

On postoperative day 5, the patient developed acute neurological deterioration. Brain CT revealed bilateral infratentorial subdural hematomas with obstructive hydrocephalus (Figure 2) as a severe complication. He underwent emergent posterior decompressive craniectomy and placement of an external ventricular drain. His recovery was favorable.

Throughout his hospitalization, he exhibited no biochemical or histological evidence of acute cellular rejection of either graft, based on protocol endomyocardial biopsies and clinical evaluation. He was discharged after five weeks.

Pathology

**Figure 3:**
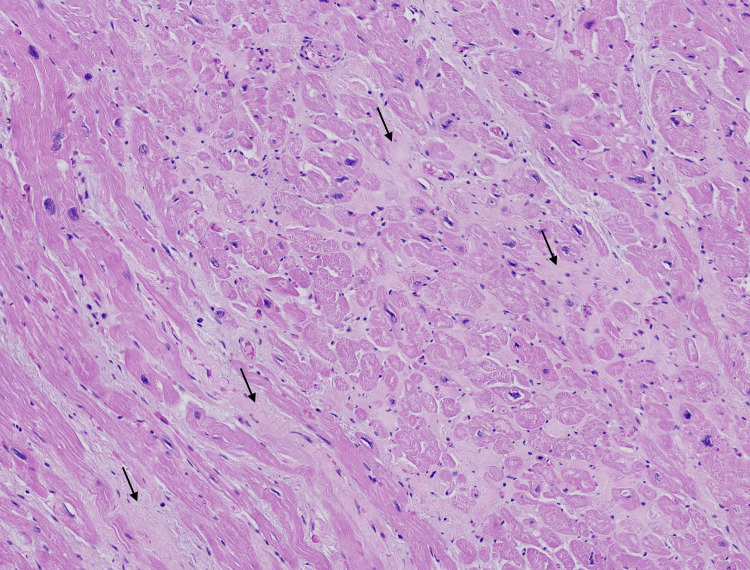
Heart tissue, low-power view Heart tissue showing focal interstitial fibrosis (arrows) and cardiomyocyte nuclear size variation. These findings are consistent with chronic myocardial remodeling and advanced dilated cardiomyopathy. H&E, 4×.

**Figure 4:**
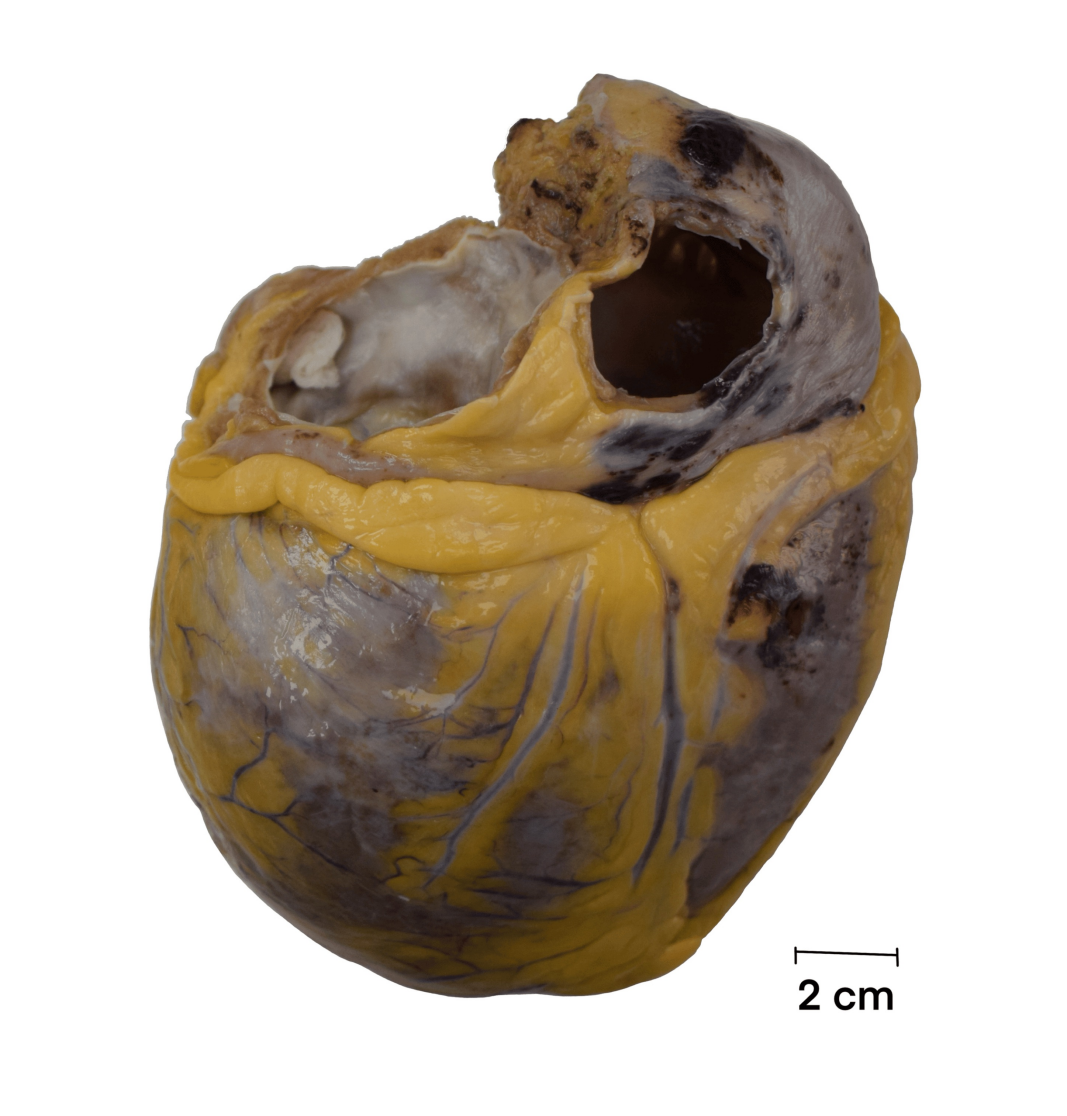
Gross appearance of the explanted heart External view of the formalin-fixed explanted heart.

**Figure 5:**
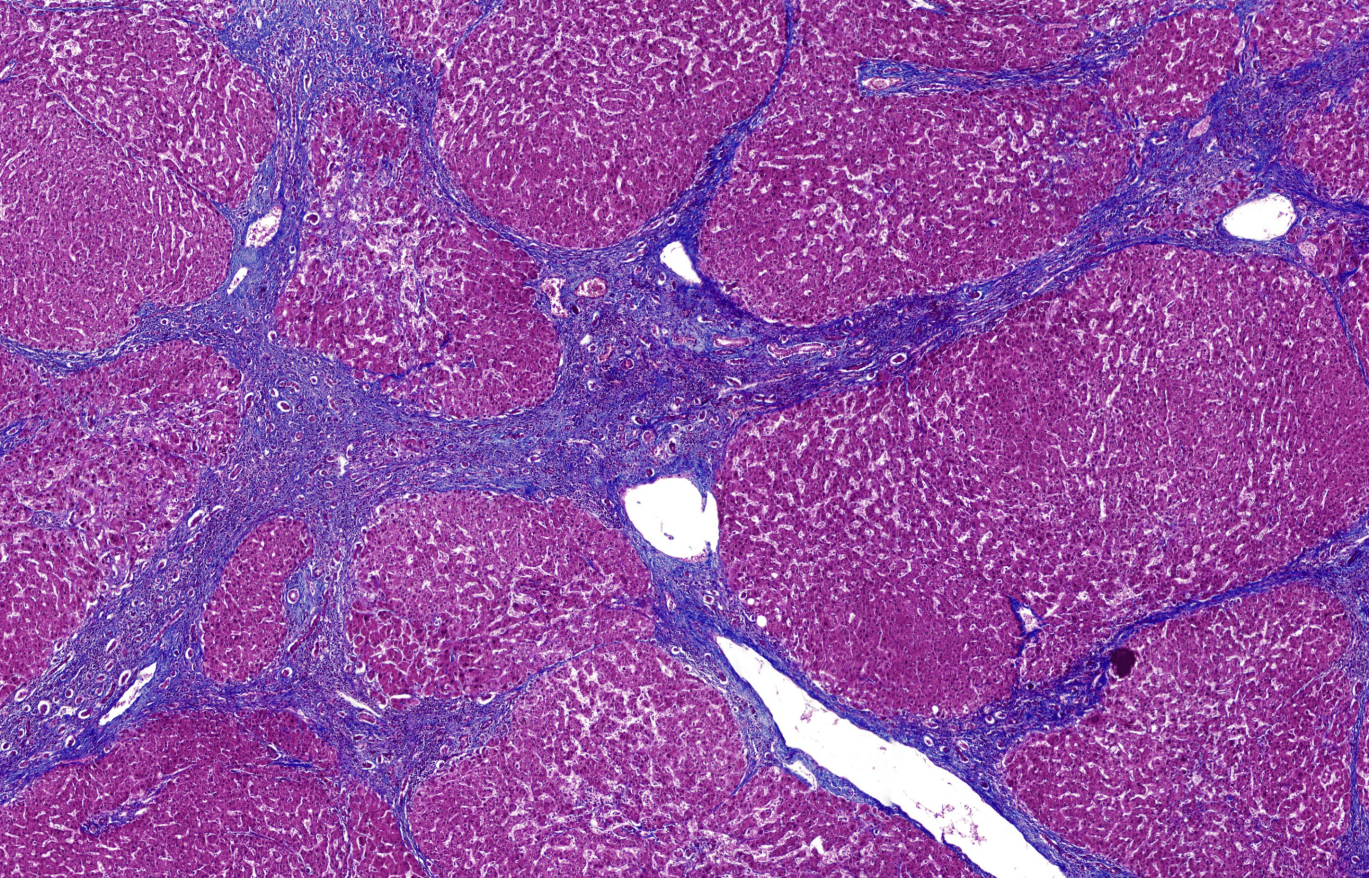
Liver tissue, low-power view Liver tissue showing diffuse architectural distortion, with rounded regenerative hepatocellular nodules separated by bridging fibrous septa, consistent with advanced cirrhosis. Masson trichrome stain, 2×.

The explanted heart showed diffuse fibrosis and myocyte hypertrophy, consistent with end-stage dilated cardiomyopathy (Figures 3, 4). The explanted liver demonstrated advanced cirrhosis with regenerative nodules and portal fibrosis without a defined etiology (Figure 5).

Follow-up

During follow-up at the UC Christus Transplant Institute, he developed biliary stenosis managed endoscopically. Cardiology and hepatology assessments confirmed excellent graft function and adequate immunosuppressive control. More than 18 months after CHLT, the patient demonstrates sustained normal graft function with good adherence to immunosuppression, NYHA I functional status, and full reintegration into work as a heavy machinery operator without limitations.

## Discussion

Although the first heart transplant in Chile was performed in 1968 [6] and the first successful liver transplant in 1985 [7], national experience with multi-organ transplantation has developed more slowly. Over the years, numerous isolated cardiac and hepatic transplants have been performed across various centers, progressively strengthening the clinical, surgical, and critical care capacity required to support more complex procedures, such as combined organ transplantation. Liver-kidney combined transplantation has been performed by different centers in Chile and Latin America [10,11], but only a few cases of CHLT have been reported. Argentina described its first case in 2006 [8], and Brazil later reported another in 2021 [9], followed by a combined heart-liver-kidney transplant in 2022 [12]. These isolated experiences reflect the limited regional adoption of such highly complex procedures. In this context, our case represents the first successful CHLT in Chile.

CHLT is indicated when cardiac and hepatic dysfunction are so interdependent that single-organ transplantation would not ensure clinical recovery. Common indications include familial amyloid polyneuropathy, cardiac cirrhosis, particularly in post-Fontan physiology, and familial hypercholesterolemia [2]. Other reported etiologies include alcoholic and cryptogenic cirrhosis, viral hepatitis, hemochromatosis, restrictive or dilated cardiomyopathies, and complex congenital heart disease [3].

The decision between isolated heart transplantation and CHLT requires a comprehensive multidisciplinary evaluation. Indications for adding liver transplantation vary among centers. They may be based on histological findings (bridging fibrosis, focal nodularity) or clinical criteria such as ascites, esophageal varices, thrombocytopenia, jaundice, splenomegaly, sarcopenia, or hepatic decompensation [13,14]. Our patient met several clinical criteria that made isolated heart transplantation unsafe.

The most widely used technique is sequential transplantation: bicaval heart transplant under cardiopulmonary bypass followed by liver transplant using the piggyback technique [4,5], as in our case. Other approaches include en bloc implantation, though they are less commonly used [1,15].

Despite its complexity, CHLT has favorable outcomes. A meta-analysis of 16 studies and 860 patients reported an overall mortality of 14.1%, with one-year survival of 85.3% and five-year survival of 71.4% [4]. Notably, CHLT recipients exhibit lower acute rejection rates of the cardiac graft compared with isolated heart transplant recipients, suggesting a potential immunomodulatory effect of the liver graft, possibly via alloantibody absorption and immune regulation [2,3,16].

Perioperative complications are common due to prolonged operative times and dual-organ ischemia. Reported ICU stays average 8-9.9 days, with total hospitalizations of 24-25.8 days [2,4]. CHLT patients may require greater transfusion support, mechanical ventilation, and circulatory support and may face a higher risk of infection. Furthermore, cirrhotic patients undergoing isolated heart transplantation experience worse postoperative outcomes, including higher reoperation rates, greater dialysis requirement, and a trend toward increased mortality [1].

Our case highlights not only the feasibility of CHLT in Chile but also the essential role of seamless multidisciplinary integration. The severe neurological complication on postoperative day 5, successfully managed by the neurosurgery team, underscores the importance of rapid, coordinated intervention in centers equipped for highly complex care.

## Conclusions

This case represents the first successful CHLT performed in Chile and demonstrates that this demanding multi-organ intervention can be safely implemented in our national context when carried out at a center with adequate multidisciplinary expertise. Beyond its technical achievement, the case highlights key elements required for successful outcomes, reinforcing the importance of careful patient selection, structured perioperative planning, and the capacity to respond effectively to severe postoperative complications. The patient’s favorable recovery and full functional reintegration underscore the central role of coordinated multidisciplinary care in achieving optimal outcomes in highly complex transplant procedures.
